# Thalamocortical functional connectivity and cannabis use in men with childhood attention-deficit/hyperactivity disorder

**DOI:** 10.1371/journal.pone.0278162

**Published:** 2022-11-28

**Authors:** Sanghyun Lee, Soon-Beom Hong

**Affiliations:** 1 Seoul National University College of Medicine, Seoul, Republic of Korea; 2 Institute of Human Behavioral Medicine, Seoul National University Medical Research Center, Seoul, Republic of Korea; Duke University Medical Center: Duke University Hospital, UNITED STATES

## Abstract

Disruptions of the cortico-striato-thalamo-cortical circuit has been implicated in both attention-deficit/hyperactivity disorder and substance use disorder. Given the high prevalence of cannabis use among patients with attention-deficit/hyperactivity disorder, we set out to investigate the relationship between the two in the thalamus. We analyzed resting-state functional magnetic resonance imaging data obtained from the Addiction Connectome Preprocessed Initiative Multimodal Treatment Study of Attention-Deficit/Hyperactivity Disorder database. Functional connectivity maps were extracted to compare thalamic connectivity among adults who had been diagnosed with attention-deficit/hyperactivity disorder during childhood according to whether or not they used cannabis. The study participants included 18 cannabis users and 15 cannabis non-users with childhood attention-deficit/hyperactivity disorder. Our results revealed that adults with attention-deficit/hyperactivity disorder who used cannabis (n = 18) had significantly decreased functional connectivity between the thalamus and parietal regions, which was particularly prominent in the inferior parietal areas, in comparison with those who did not use cannabis (n = 15). Left thalamic functional connectivity with the inferior parietal and middle frontal areas and right thalamic functional connectivity with the inferior parietal and superior frontal areas were increased in non-users of cannabis with attention-deficit/hyperactivity disorder compared with a local normative comparison group (n = 7). In conclusion, adults with a childhood history of attention-deficit/hyperactivity disorder who do not use cannabis often have relatively stronger thalamoparietal and thalamofrontal connectivity, which may help reduce the risk of cannabis use.

## Introduction

Attention-deficit/hyperactivity disorder (ADHD) is a neurodevelopmental disorder that is characterized by inattention, hyperactivity, and impulsivity. It is one of the most common mental disorders among children, with a prevalence of approximately 5% [1]. Up to 65% of patients with ADHD have symptoms that persist into adulthood [2], resulting in long-lasting psychosocial impairment [3,4]. Patients with ADHD show increased reward-seeking behavior [5] and are at increased risk of substance use disorder [6]. Moreover, approximately 23% of patients with substance use disorder have been found to have ADHD [7]. Cannabis is one of the most commonly used illegal or controlled substances worldwide [8], with high rates of use in adolescence and early adulthood [9]. One study found that children with ADHD had almost three times higher odds of having ever used marijuana and were 1.5 times more likely to become cannabis-dependent than those without ADHD [6]. In another study, the rate of cannabis use during 8 years of follow-up was significantly higher in adolescents who had a diagnosis of ADHD in childhood (32%) than in their typically developing counterparts (24%) [10]. More specifically, increased odds of lifetime cannabis use (odds ratio = 2.78) and diagnosis with cannabis use disorder during young adulthood (odds ratio = 1.51) were reported for people diagnosed with ADHD in childhood according to meta-analyses [6,11].

The thalamus is an important component of the cortico-striato-thalamo-cortical circuit, disruption of which has been implicated in ADHD [12]. Preschool-aged children with ADHD have reduced subcortical volumes, including in the thalamus [13], although this finding was not replicated in a large sample of older children [14]. Other studies found that adults with a diagnosis of ADHD in childhood had decreased brain activity, including in the thalamus, during tasks involving inhibitory control and cognitive flexibility [15], which may be associated with substance use disorder [16–18]. Moreover, resting-state functional connectivity between the thalamus and putamen is significantly greater in children with ADHD than in typically developing children [19,20]. The thalamus may play an important role in addiction, especially in terms of reduced response inhibition and enhanced salience to drug cues [21]. Decreased thalamic resting-state functional connectivity has been observed in smokers, long-term abstinent alcoholics, and young adults with untreated cannabis use disorder [22–24]. Furthermore, thalamic volumes have been found to be bilaterally reduced in users of synthetic cannabinoids [25]. Methylphenidate, which is one of the medications most commonly used in ADHD, significantly increases resting-state connectivity between the thalamus and the cerebellum in both cannabis abusers and healthy controls, whereas methylphenidate-induced increases in thalamic metabolism were blunted in participants with cannabis use disorder in comparison with controls [26].

In summary, both ADHD and cannabis use may be associated with resting-state functional connectivity of the thalamus. However, little is known about the role of functional thalamic connectivity in the increased risk of cannabis use in patients with ADHD, and few studies have addressed this issue. Therefore, we aimed to explore the relationship between ADHD and cannabis use in the brain, with a special focus on the thalamus. Based on the findings of previous studies [22–24,26], we hypothesized that thalamic connectivity would be decreased in cannabis users with a childhood diagnosis of ADHD. In this study, we investigated resting-state functional thalamic connectivity in adults who had been diagnosed with ADHD in childhood according to their cannabis use status.

## Methods

### Study design and participants

The Multimodal Treatment Study of ADHD (MTA) was initiated as a randomized clinical trial funded by the National Institute of Mental Health, and the participants were subsequently followed up in a longitudinal study. In this study, we analyzed resting-state functional magnetic resonance imaging (rs-fMRI) data obtained from the publicly available Addiction Connectome Preprocessed Initiative (ACPI) MTA database (http://fcon_1000.projects.nitrc.org/indi/ACPI/html/index.html), which is supported by the National Institute on Drug Abuse. The complete dataset consisted of MRI scans and phenotypic information collected from six different sites. The phenotypic information released from the ACPI database included age, sex, intelligence quotient, data collection site, handedness, ethnicity, education level, being a smoker or not, and whether currently taking ADHD medication, in addition to the group assignment for ADHD and marijuana use. Other potentially important details such as anxiety, depression, use of substances other than marijuana and tobacco, or treatment assignment in the original MTA were not available from the released dataset.

In the original MTA, participants met the Diagnostic and Statistical Manual of Mental Disorders, fourth edition, criteria for ADHD combined type, based on the Diagnostic Interview Schedule for Children (DISC), parent report, which was supplemented with up to two symptoms reported by children’s teachers based on the Swanson, Nolan and Pelham-IV (SNAP-IV) questionnaire when falling just below the DISC diagnostic threshold [27]. In addition, age-matched and sex-matched classmates of children with ADHD were recruited as a local normative comparison group (LNCG) [28]. In the ACPI dataset, a total of 129 study participants were classified into four groups: ADHD marijuana users (group 1, 39 males and 3 females), ADHD marijuana non-users (group 2, 34 males and 12 females), LNCG marijuana users (group 3, 17 males and 3 females), and LNCG marijuana non-users (group 4, 14 males and 7 females). Participants were classified as marijuana users if they used marijuana once per month or more and non-users if they had used marijuana fewer than four times during the past year, which was identified based on self-reported cannabis use using the Substance Use Questionnaire and Substance Use Recency Questionnaire [29]. In line with the high male predominance in ADHD and in cannabis use [1,30], there were significant differences in the male-to-female ratio among groups, which could have had a serious confounding influence on our findings; therefore, we only included male participants in our analysis. Limited information was available on the scanning parameters via the ACPI database; however, more information can be found in a report by the data collectors (i.e., the MTA Neuroimaging Group) [29].

Informed written consent was obtained from all participating families in the original MTA [27]. This study used deidentified data and was granted exemption from a review by the Institutional Review Board at the Seoul National University Hospital. All methods were performed in accordance with the relevant guidelines and regulations.

### Image processing

We used rs-fMRI data that had been preprocessed using Advanced Normalization Tools (ANTs) software (http://stnava.github.io/ANTs/) and released by the ACPI. The ACPI provides four different versions of the preprocessed data according to motion correction (with or without scrubbing) and nuisance correction (with and without global signal regression). To ensure the robustness of our findings, we used all four versions of the preprocessed datasets in our analyses. All steps in the processing pipeline are described at http://fcon_1000.projects.nitrc.org/indi/ACPI/html/preproc.html.

Given that head motion in the scanner has been a serious confounder in rs-fMRI studies [31,32], we excluded participants who had fewer than 100 scans after scrubbing. This resulted in sample sizes of 18, 15, 9, and 7 male participants, respectively, in groups 1, 2, 3, and 4. Based on our hypothesis and interest in the implication of thalamic connectivity in cannabis use among individuals with ADHD, and considering the small sample size of the other two groups representing non-ADHD participants, a comparison of thalamic functional connectivity between the first two groups was the focus in this study. Among the 25 female participants included in the ACPI dataset, the exclusion of participants who had fewer than 100 scans after scrubbing resulted in sample sizes of 0, 7, 2, and 3 female participants, respectively, in groups 1, 2, 3, and 4. Thus, we decided to focus on male participants as including both sexes in the analysis would have caused a severe imbalance in the male-to-female ratio, and the absence of female participants in group 1, in particular, limited our ability to match the number of each sex among groups.

This final sample was equally applied to the analysis of all four versions of the preprocessed datasets. Regarding the preprocessed data without scrubbing, the number of rs-fMRI measurements was either 180 or 128 depending on the data collection site. We discarded some of the later scans from the 180-scan data and analyzed the first 128 scans for all participants.

We obtained whole-brain functional connectivity maps of the left and right thalamic seed regions using the Data Processing & Analysis of Brain Imaging (DPABI) tool [33] and the Automated Anatomical Labeling atlas [34], which was resampled to the space of functional images.

### Data analysis

Between-group differences in descriptive statistics were examined using the Student’s *t*-test and chi-squared or Fisher’s exact test for continuous and categorical variables, respectively. Statistical tests were performed using SPSS (version 25.0; IBM, Armonk, NY), and the results were reported with a significance threshold of uncorrected *p* < 0.05.

We compared thalamic functional connectivity maps between ADHD marijuana users and non-users. We set the cluster-forming height threshold at *p* < 0.001 uncorrected, and we used a family-wise error (FWE)-corrected cluster-defining threshold of *p* < 0.025, which was Bonferroni-adjusted (0.05/2) because we explored the left and right thalamic seeds separately. The analyses were performed using Statistical Parametric Mapping (SPM), which is based on MATLAB (MathWorks).

## Results

### Participant characteristics

The study participants included 18 cannabis users and 15 cannabis non-users with childhood ADHD. There was no significant difference in age, intelligence quotient, data collection site, handedness, ethnicity, or the proportion of smokers between the two groups (Table 1).

**Table 1:** Characteristics of adults with childhood ADHD.

Demographics	Cannabis users (n = 18)		Cannabis non-users (n = 15)		*p*-value
**Age (years), mean (SD)**	23.94	1.30	24.27	1.33	0.665
**Sex (male), n (%)**	18	100.0	15	100.0	N/A
**IQ, mean (SD)**	104.28	12.52	111.00	14.78	0.548
**Handedness (right), n (%)**	16	88.9	11	73.3	0.375
**Smoker, n (%)**	10	55.6	5	33.3	0.202
**Currently taking ADHD medication (no), n (%)**	16	88.9	14	93.3	1.000
**Education (high school equivalent or lower), n (%)**	16	88.9	6	40.0	0.003
**Ethnicity (Caucasian), n (%)**	10	55.6	11	73.3	0.290

ADHD, attention-deficit/hyperactivity disorder; IQ, intelligence quotient; SD, standard deviation.

### Between-group differences in functional connectivity

**Fig 1:**
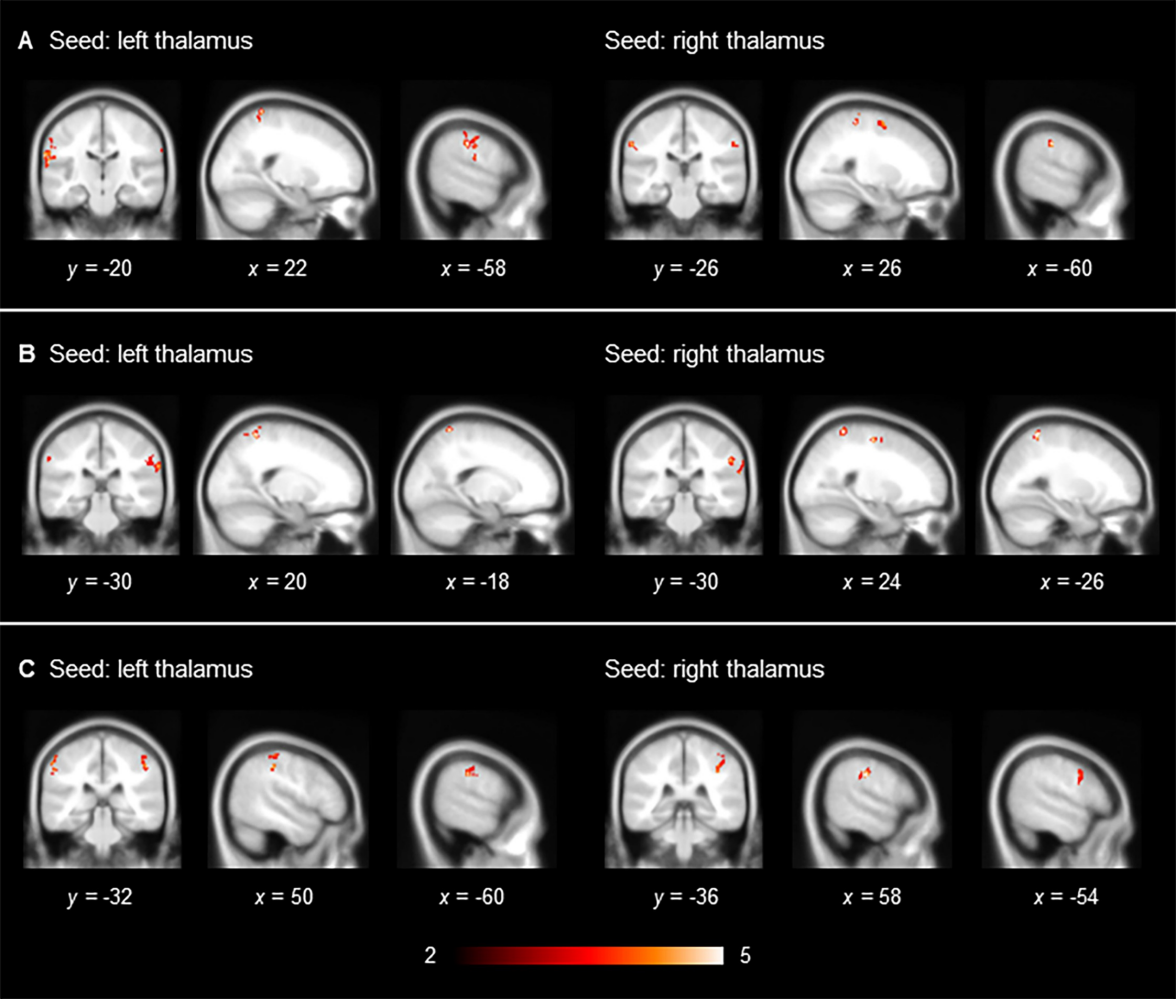
Significant increase in thalamic functional connectivity in adult cannabis non-users with a childhood diagnosis of ADHD compared with their counterparts who were cannabis users. (A) Scrubbing (+), global signal regression (+); (B) scrubbing (−), global signal regression (+), and (C) scrubbing (−), global signal regression (−). Results are displayed at a family-wise error-corrected cluster-defining threshold of *p* < 0.025.

Compared with non-users, cannabis users showed a significant decrease in functional connectivity between the thalamus and the parietal regions in particular, which was particularly prominent in the inferior parietal areas, including the supramarginal gyrus (Table 2, Fig 1). These findings were largely consistent across the different preprocessing strategies. There was greater functional connectivity between the thalamus and the cuneus and calcarine gyrus in cannabis users than in non-users, which was not replicated across different sets of preprocessed data (Table 2).

**Table 2:** Regional differences in thalamic functional connectivity between adult cannabis users and non-users, with childhood ADHD.

	Seed: left thalamus						Seed: right thalamus					
Brain region	Peak coordinates (x, y, z)			Statistics		*p*-value	Peak coordinates (x, y, z)			Statistics		*p*-value
				K_E_	Z					K_E_	Z	
**Cannabis users < non-users**												
**Scrubbing (+), GSR (+)**												
**Supramarginal**	-58	-26	36	75	4.41	0.000	52	-30	34	44	4.10	0.016
**Precentral**	24	-14	56	51	4.30	0.007	-54	6	34	44	4.18	0.016
**Superior temporal**	64	-16	6	57	4.43	0.003	58	-6	-10	42	4.48	0.021
**Superior parietal**	22	-46	72	42	4.32	0.023						
**Superior frontal**							28	-4	60	55	3.88	0.004
**Postcentral**	-62	-20	16	65	4.20	0.001						
**Scrubbing (+), GSR (-)**												
**Supramarginal**	-60	-28	36	73	4.69	0.001						
**Inferior parietal**	44	-40	56	67	3.92	0.002						
**Postcentral**	32	-24	46	45	4.59	0.023						
**Scrubbing (-), GSR (+)**												
**Supramarginal**	66	-30	28	87	4.06	0.000	66	-24	24	52	3.85	0.006
**Supramarginal**							54	-30	36	48	4.08	0.009
**Precentral**	24	-14	56	57	5.11	0.003	24	-14	58	76	4.30	0.000
**Precentral**							-56	8	36	48	4.05	0.009
**Postcentral**	64	-14	34	57	3.90	0.003	24	-46	68	109	4.49	0.000
**Superior parietal**	20	-50	64	79	4.53	0.000	-26	-50	64	54	5.30	0.004
**Inferior parietal**	-56	-18	40	80	4.16	0.000						
**Inferior parietal**	-54	-34	48	61	4.16	0.002						
**Supplementary motor area**	4	-4	56	57	3.97	0.003						
**Scrubbing (-), GSR (-)**												
**Supramarginal**	-60	-28	36	88	4.57	0.000	58	-22	36	57	4.64	0.004
**Supramarginal**	50	-32	44	105	4.03	0.000	40	-36	44	44	3.80	0.022
**Precentral**	24	-14	56	45	4.54	0.021	24	-14	58	65	4.25	0.002
**Postcentral**	32	-24	46	87	4.04	0.000						
**Inferior parietal**	-56	-34	50	65	4.09	0.002						
**Cannabis users > non-users**												
**Scrubbing (+), GSR (+)**												
**Cuneus**	4	-86	18	128	4.65	0.000						
**Scrubbing (-), GSR (+)**												
**Calcarine**	10	-86	6	239	4.36	0.000						

ADHD, attention-deficit/hyperactivity disorder; GSR, global signal regression; K_E_, cluster extent in voxels. Positive and negative x coordinates indicate right and left hemispheres, respectively. Results are displayed at a family-wise error-corrected cluster-defining threshold of *p* < 0.025. A p-value < 0.00625 survives an additional Bonferroni correction for the number of preprocessing pipelines (0.025/4).

### Additional tests on the direction of change in functional connectivity

**Fig 2:**
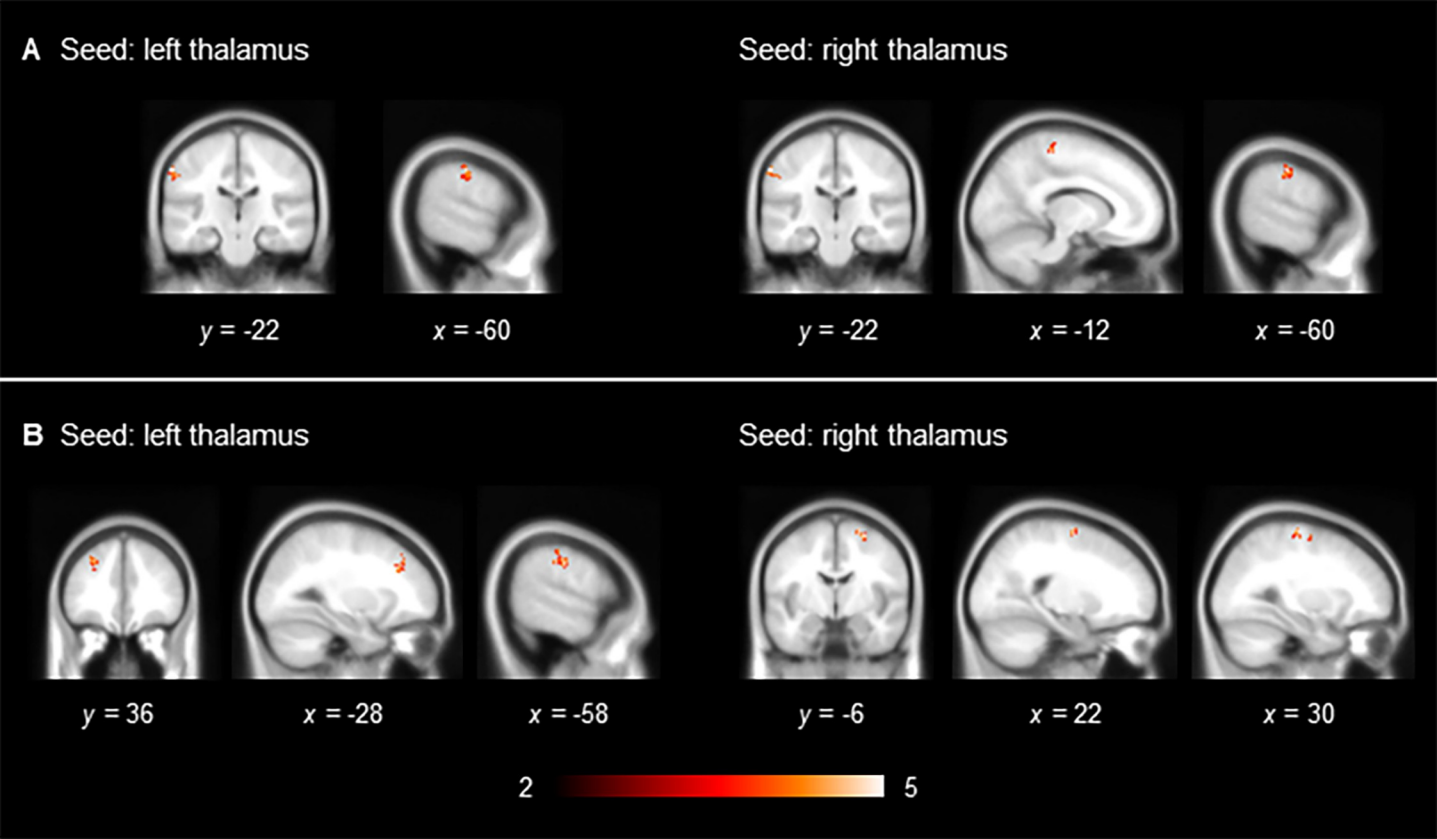
Significant increase in thalamic functional connectivity in adult cannabis non-users with a childhood diagnosis of ADHD compared with cannabis non-users without a childhood diagnosis of ADHD. (A) Scrubbing (+), global signal regression (−) and (B) scrubbing (−), global signal regression (−). Results are displayed at a family-wise error-corrected cluster-defining threshold of *p* < 0.025.

To explore whether the observed between-group difference was due to a decrease in connectivity among cannabis users or to an increase in connectivity among cannabis non-users, we performed additional analyses to compare their thalamic functional connectivity with that in the LNCG without childhood ADHD who were cannabis non-users (n = 7). There were no significant differences in descriptive parameters between the three groups, except in the proportion of smokers between ADHD cannabis users and non-users in the LNCG (S1 Table). We found significantly increased functional connectivity between the left thalamus and brain regions that encompassed the inferior parietal and middle frontal areas and significantly increased functional connectivity between the right thalamus and brain regions encompassing the inferior parietal and superior frontal areas in non-users with ADHD compared with those in the LNCG (Table 3, Fig 2). There was no significant decrease in thalamic connectivity in ADHD cannabis non-users and no significant difference in thalamic connectivity between ADHD cannabis users and the LNCG.

**Table 3:** Regional differences in thalamic functional connectivity among adult cannabis non-users, with and without childhood ADHD.

	Seed: left thalamus						Seed: right thalamus					
Brain region	Peak coordinates (x, y, z)			Statistics		*p*-value	Peak coordinates (x, y, z)			Statistics		*p*-value
				K_E_	Z					K_E_	Z	
**ADHD > LNCG**												
**Scrubbing (+), GSR (-)**												
**Supramarginal**	-62	-22	44	61	4.28	0.002	-62	-22	44	75	4.54	0.000
**Scrubbing (-), GSR (-)**												
**Precentral**	-20	-22	66	56	4.45	0.003	30	-20	66	46	3.98	0.008
**Superior frontal**							22	-6	64	40	4.30	0.019
**Middle frontal**	-26	28	34	58	4.45	0.002						
**Supramarginal**							-58	-24	40	73	4.05	0.000
**Supramarginal**							66	-24	34	39	4.03	0.022
**Postcentral**	-58	-20	34	86	4.14	0.000						

ADHD, attention-deficit/hyperactivity disorder; LNCG, local normative comparison group; GSR, global signal regression; K_E_, cluster extent in voxels. Positive and negative x coordinates indicate right and left hemispheres, respectively. Results are displayed at a family-wise error-corrected cluster-defining threshold of *p* < 0.025. A p-value < 0.00625 survives an additional Bonferroni correction for the number of preprocessing pipelines (0.025/4).

## Discussion

We found increased resting-state functional connectivity between the thalamus and the inferior and superior parietal cortices in particular as well as the superior and middle frontal cortices in adult cannabis non-users with a childhood diagnosis of ADHD.

The thalamus has been hypothesized to contribute to an increased addiction risk by enhancing responses to salient drug cues and attenuating the ability to inhibit these responses [21]. Salience processing refers to the identification of important external and/or interoceptive stimuli. The anterior insula and anterior midcingulate cortex are the central components of the brain’s salience network, and distributed brain regions, including the inferior parietal cortex and basal ventromedial nucleus of the thalamus, also participate in this large-scale brain network [35,36]. Our present findings of increased resting-state functional connectivity between the thalamus and inferior parietal cortex in cannabis non-users may thus be regarded in the context of salience processing toward drug cues. However, the inferior parietal lobe is also a convergence zone for different mental abilities, including language, attention, mathematical calculation, and social cognition [37]. Furthermore, long-term heavy cannabis use is associated with impaired working memory [38], which may be related to damage to the superior parietal cortex [39]. In one study, even after prolonged abstinence, brain processing efforts were greater in cannabis users than in non-users during inhibitory control, as evidenced by an increased blood-oxygen-level-dependent response in the frontal cortex as well as inferior and superior parietal cortices [40]. In summary, the increased thalamoparietal connectivity observed in cannabis non-users may reflect a better ability to control impulsive responses to drug cues [41], which would in turn facilitate more appropriate attribution of salience to behaviorally relevant stimuli other than cannabis (Table 4).

**Table 4:** Summary of findings and possible implications.

Comparison samples	Findings	Possible implications
Cannabis users and non-users with childhood ADHD	Increased thalamoparietal connectivity in non-users	Better ability to control impulsive responses to drug cues, accompanied by more appropriate attribution of salience to behaviorally relevant stimuli other than cannabis
Cannabis non-users with childhood ADHD and LNCG without childhood ADHD	Increased thalamoparietal connectivity in non-users	Possible improvement in the ability to control impulsive responses to drug cues, accompanied by more appropriate attribution of salience to behaviorally relevant stimuli other than cannabis
Cannabis non-users with childhood ADHD and LNCG without childhood ADHD	Increased thalamofrontal connectivity in non-users	Possible improvement in inhibitory control, which helps inhibit responses to drug cues and reduce the frequency of cannabis use
Cannabis users with childhood ADHD and LNCG without childhood ADHD	No significant difference in thalamic connectivity	N/A

ADHD, attention-deficit/hyperactivity disorder; LNCG, local normative comparison group.

The increased thalamoparietal connectivity in cannabis non-users with childhood ADHD was not only evident in the comparison with regular users of cannabis who had been diagnosed with ADHD but also in the comparison with the LNCG. Moreover, cannabis non-users with childhood ADHD showed increased thalamofrontal connectivity. The thalamus appears to mediate inhibitory control via its connectivity with the prefrontal cortex [42], and age-related progressive strengthening of thalamofrontal functional connectivity was observed in a sample of healthy children and adults [43]. Although patients with ADHD do not generally outgrow the disorder by adulthood, intermittent periods of remission can be expected in most cases [44]. Moreover, there has been a study showing that functional connectivity in the brains of adults whose inattentive symptoms had resolved did not differ significantly from that of their never-affected peers [45]. Therefore, adults who do not regularly use cannabis but have a past history of ADHD perhaps constitute a subgroup that provisionally attains relatively better thalamofrontal and thalamoparietal function, which can help inhibit responses to drug cues and thus reduce the frequency of cannabis use (Table 4).

Other brain regions that show reduced functional connectivity with the thalamus in cannabis users have also been implicated in addiction in the literature. Cannabis users demonstrated significantly less activation of the precentral and postcentral regions during a finger-sequencing task compared with non-users [46,47]. Furthermore, the persistence of ADHD was associated with smaller precentral and postcentral cortical thickness, while the early onset of cannabis use in patients with ADHD was associated with greater postcentral cortical thickness [48]. Altered activation of the primary somatosensory cortex was associated with risky decision-making in patients with substance use disorder [49]. We found large symmetric differences in thalamic connectivity in both hemispheres, which is consistent with the almost exact mirror symmetry of resting-state connectivity between the thalamus and cortex in healthy young adults [50].

Using the ACPI MTA data, Kelly and colleagues reported significant main effects of cannabis use history in two out of the nine large-scale intrinsic connectivity networks: the right superior temporal sulcus within the default network and the left fusiform gyrus within the lateral visual network both exhibited stronger intrinsic functional connectivity in cannabis users relative to non-users [29]. The nine large-scale intrinsic connectivity networks examined in this report included one network predominantly involving subcortical regions such as the amygdala, putamen, and thalamus; however, no significant findings were observed in the analysis using the predominantly subcortical network. The present work differs from that of Kelly and colleagues in that we specifically focused on the functional connectivity of the thalamus, based on a seed-to-whole brain approach using the thalamus as the seed, and found significant difference in thalamic connectivity between ADHD cannabis users and non-users. To further explore whether this difference was due to a decrease in connectivity among cannabis users or to an increase in connectivity among cannabis non-users, we performed additional analyses to compare their thalamic functional connectivity with that in the LNCG without childhood ADHD who were cannabis non-users. The only difference found in these subsequent analyses was increased bilateral thalamic functional connectivity in non-users with ADHD, and no significant difference in thalamic connectivity was observed between ADHD cannabis users and the LNCG. Albeit with different methods, Kelly and colleagues also did not observe a decrease in connectivity among cannabis users within the nine large-scale intrinsic connectivity networks tested, which is in line with the interpretation of our findings as an increase in connectivity among ADHD cannabis non-users rather than a decrease in connectivity among ADHD cannabis users.

This study has several limitations. In the original MTA, four different treatments were randomly assigned to children with ADHD. However, no information was available on which participant received which treatment, whether the participant continued treatment during a period of extended follow-up, or the presence or absence of ADHD at the time of scanning. For example, it would also be interesting to examine whether thalamocortical connectivity displays any variations according to the level of compliance with medication. In addition, no information was available on the type of stimulant medication used for each participant (e.g., methylphenidate or dextroamphetamine) in the ACPI database. Moreover, behavioral measures of ADHD symptoms and cannabis use were not included in the dataset. Therefore, we were not able to explore the functional implications of altered thalamocortical connectivity. Considering that the original MTA collected a comprehensive set of clinical information, integrating those data into the ACPI database would help further research. In particular, more information is needed about cannabis use, such as the type of cannabis used, route of administration, as well as date of last use. For example, we were not able to verify if any participant had recently consumed cannabis before imaging acquisition, which is an important limitation considering that cannabinoids may affect brain functioning for 28 days after last use [51]. In addition, information about cannabis use was collected based on self-report, limiting data reliability. Moreover, no information was available on other psychiatric or substance use disorders in the ACPI database. Considering that ADHD is frequently comorbid with other mental disorders, absence of such information is an important limitation of this study. Finally, the sample size was small, and we only included men in our analysis because of the small number of women, which limits the generalizability of our findings. However, it is noteworthy that a high male predominance is among the typical characteristics of the population of interest [1,30]. The exclusion of participants with excessive head motion resulted in a further reduction of the study sample size, but may also contribute to the strength of the study. As the current study was based on an existing dataset, we were unable to alter the sample size, and despite the small sample size, we considered that the ACPI dataset was worthy of research as it contains unique data collected over a long time frame that would allow investigation of the link between prior childhood ADHD and later marijuana use in adulthood. Notably, despite the small sample size, our findings were observed at a relatively strong statistical significance level in terms of cluster size and p-value. Many of the findings survived a stringent Bonferroni correction for two thalamic seeds tested as well as four different preprocessing pipelines (*p* < 0.00625; 0.05/8). We expect that the current findings will provide an initial basis for further studies with a larger collection of data.

## Conclusion

In conclusion, adults with a childhood diagnosis of ADHD who did not frequently use cannabis showed increased thalamoparietal connectivity compared with those who had ADHD in childhood and were regular users of cannabis. The increased thalamoparietal connectivity was replicated and increased thalamofrontal connectivity was observed in comparison with their peers who were cannabis non-users without a history of childhood ADHD. These findings support the hypothesis that the thalamus may be involved in cannabis use, at least in individuals with a past history of ADHD. Considering that individuals with ADHD are at a higher risk for other substance use, it would be interesting to examine if the implications of thalamic connectivity reported herein extend to other substance use. In addition, it would be interesting to examine if improvement in ADHD symptoms renders some protective effect against addiction, and whether an increase in thalamoparietal and/or thalamofrontal connectivity plays a role in this protective effect. Further studies are needed to examine thalamocortical connectivity in a larger sample of participants with ADHD and/or substance use disorder and its functional implications in response inhibition and salience to drug cues.

## Supporting information

S1 Table Characteristics of participants without ADHD. (DOCX)
