# Supplementary material for: Thalamocortical functional connectivity and cannabis use in men with childhood attention-deficit/hyperactivity disorder
Source: PLoS One. 2022 Nov 28;17(11):e0278162. doi: 10.1371/journal.pone.0278162 (PMC9704667; doi:10.1371/journal.pone.0278162)
Supplement: S1 Table — (DOCX) [file pone.0278162.s001.docx]

Supplemental Table 1. Characteristics of participants without ADHD

|  | LNCG cannabis non-users (n=7) | | p-value* | p-value^†^ |
| --- | --- | --- | --- | --- |
| Age (years), mean (SD) | 23.29 | 1.70 | 0.268 | 0.387 |
| Sex (male), n (%) | 7 | 100.0 | N/A | N/A |
| IQ, mean (SD) | 115.57 | 21.30 | 0.104 | 0.289 |
| Handedness (right), n (%) | 5 | 71.4 | 0.548 | 1.000 |
| Smoker, n (%) | 0 | 0.0 | 0.020 | 0.135 |
| Currently taking ADHD medication (no), n (%) | 7 | 100.0 | 1.000 | 1.000 |
| Education (high school equivalent or lower), n (%) | 4 | 57.1 | 0.113 | 0.652 |
| Ethnicity (Caucasian), n (%) | 3 | 42.9 | 0.673 | 0.343 |

ADHD, attention-deficit/hyperactivity disorder; IQ, intelligence quotient; LNCG, local normative comparison group; SD, standard deviation. *ADHD cannabis users vs. non-users in the LNCG. ^†^ADHD cannabis non-users vs. LNCG non-users.
